# Preoperative transcatheter arterial chemoembolization and prognosis of patients with solitary large hepatocellular carcinomas (≥5 cm): Multicenter retrospective study

**DOI:** 10.1002/cam4.5529

**Published:** 2022-12-20

**Authors:** Ali Mo, Qiao Zhang, Feng Xia, Zhiyuan Huang, Shasha Peng, Wenjing Cao, Hongliang Mei, Li Ren, Yang Su, Hengyi Gao, Weiqiang Chen

**Affiliations:** ^1^ Guangdong Medical University Guangdong China; ^2^ Department of Hepatobiliary Surgery Zhongshan People's Hospital Zhongshan City China; ^3^ Department of Hepatic Surgery Center Tongji Hospital of Tongji Medical College of Huazhong University of Science and Technology Wuhan China; ^4^ Department of Hepatobiliary and Pancreatic Surgery,Huangshi Central Hospital of Edong Healthcare Group Hubei Polytechnic University Huangshi China; ^5^ Southern Medical University Graduate School Guangzhou City China; ^6^ General Surgery General Hospital of Central Theater Wuhan China; ^7^ Hepatobiliary Pancreatic Surgery Qinghai University Affiliated Hospital Xining China; ^8^ Department of Hepatobiliary Surgery Renmin Hospital of Wuhan University Wuhan China; ^9^ Department of Hepatobiliary and Pancreatic Surgery Shenzhen Longhua District People's Hospital Shenzhen City China

**Keywords:** hepatectomy, hepatocellular carcinoma, recurrence survival, transcatheter arterial chemoembolization

## Abstract

**Objectives:**

Large hepatocellular carcinoma (LHCC) is prone to short‐term recurrence and poor long‐term survival after hepatectomy, and there is still a lack of effective neoadjuvant treatments to improve recurrence‐free survival (RFS) and overall survival (OS). We retrospectively analyzed the efficacy of preoperative transcatheter arterial chemoembolization (TACE) in solitary LHCC (≥5 cm).

**Materials and Methods:**

A multicenter medical database was used to analyze preoperative TACE's effects on RFS, OS, and perioperative complications in patients with solitary LHCC who received surgical treatment from January 2005 to December 2015. The patients were divided into Group A (5.0–9.9 cm) and Group B (≥10 cm), with 10 cm as the critical value, and the effect of preoperative TACE on RFS, OS and perioperative complications was assessed in each subgroup.

**Results:**

In the overall population, patients with preoperative TACE had better RFS and OS than those without preoperative TACE. However, after stratifying the patients into the two HCC groups, preoperative TACE only improved the survival outcomes of patients with Group B (≥10 cm). Multivariate Cox‐regression analysis showed that lack of preoperative TACE was an independent risk factor for RFS and OS in the overall population and in Group B but not in Group A.

**Conclusions:**

Preoperative TACE is beneficial for patients with solitary HCC (≥10 cm).

## Graphical abstract

First, preoperative imaging evaluation of HCC patients was performed to evaluate tumor size and location.Second, whether or not a patient should receive TACE before surgical resection is determined according to tumor diameter.Finally, with the assistance of interventional equipment, the blood vessels of nutritional tumors are identified and embolized.

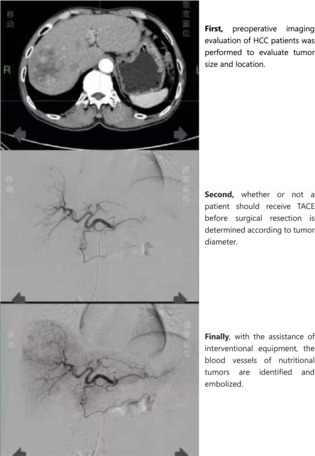

## 1 INTRODUCTION

Hepatocellular carcinoma (HCC) is the sixth most common malignancy worldwide and the third leading cause of cancer‐related death.^1^ For HCC with a relatively early stage of disease, hepatectomy has been considered a radical treatment that can achieve a good survival prognosis.^2^, ^3^, ^4^ However, the tumor is highly prone to recurrence after hepatectomy, resulting in the patient's death, particularly in HCC patients with larger tumor diameters and microvascular invasion (MVI).^5^, ^6^, ^7^ Therefore, we should take appropriate measures to reduce recurrence and improve the overall survival (OS) of patients.^8^

Transcatheter arterial chemoembolization (TACE), as an effective local treatment, can improve the OS of patients with unresectable HCC, and thus is often used for the treatment of advanced HCC.^9^, ^10^, ^11^, ^12^, ^13^ A large number of studies in the past have confirmed that postoperative TACE can reduce recurrence and prolong OS of patients,^14^, ^15^, ^16^, ^17^ and can TACE also be used as a neoadjuvant treatment for HCC? There is still controversy on the benefits of using TACE as a neoadjuvant therapy.^18^, ^19^, ^20^, ^21^, ^22^, ^23^, ^24^, ^25^, ^26^, ^27^, ^28^, ^29^, ^30^, ^31^, ^32^, ^33^, ^34^, ^35^, ^36^, ^37^, ^38^, ^39^, ^40^, ^41^ Some scholars have proposed that preoperative TACE is not beneficial for all of patients with HCC, and whether it can improve the long‐term survival mainly depends on the diameter of the tumor.^17^, ^18^, ^20^, ^25^, ^27^ To explore whether the efficacy of preoperative TACE depends on the tumor diameter, we used a multicenter database to stratify patients according to tumor diameter and, for the first time, explored the efficacy of preoperative TACE in patients with large hepatocellular carcinoma (LHCC) in different tumor diameter groups.

## 2 MATERIALS AND METHODS

### 2.1 Patient population

This study was based on patients who underwent curative resection of HCC in Zhongshan People's Hospital, Hubei Xiaogan Central Hospital, People's Hospital of Wuhan University, Qinghai University Affiliated Hospital, Huangshi Central Hospital, and Wuhan Tongji Hospital from January 2005 to December 2015. Inclusion criteria (1) a solitary HCC with tumor diameter ≥5 cm; (2) a postoperative pathological diagnosis of HCC; (3) no extrahepatic metastasis; (4) no radiologic evidence of invasion into the major portal/hepatic vein branches; (5) radical resection of HCC (R0), that is, no residual tumor tissue under direct observation or microscopy; (6) no previous treatment of HCC. Exclusion criteria: (1) younger than 18 years old; (2) poor liver function with Pugh‐Child Class C; (3) missing prognosis and follow‐up information. The ethics committees of the six medical centers approved the study, and the study complied with the Helsinki Declaration and local laws.

### 2.2 Data collection

All patients underwent contrast‐enhanced computed tomography (CT), magnetic resonance imaging (MRI), or chest X‐ray scanning upon admission to the hospital. Preoperative Information on baseline patient characteristics includes age, sex, diabetes mellitus, etiology of liver diseases, cirrhosis, Child‐Pugh grade, platelets count, international normalized ratio (INR), alpha‐fetoprotein (AFP) level, the presence or absence of postoperative adjuvant TACE, maximum tumor size, MVI, satellite nodules, tumor differentiation, and tumor capsule. Continuous variables, such as age, are transformed into binary variables according to recognized cut‐off values or upper and lower lines of normal values.^8^, ^18^, ^42^ Anatomic resection refers to the resection of one or more adjacent hepatic sections along the hepatic vasculature and includes segmentectomy, subsegmentectomy, sectionctomy, and hemihepatectomy. Non‐anatomic resection is defined as local resection or enucleation regardless of the anatomical segment or section of the lobar anatomy.^43^, ^44^

### 2.3 Preoperative TACE

Considering that this was a retrospective study, the decision to use TACE prior to surgery was left to the discretion of the treating surgeon and the patient at that time. The patient was placed supine, locally disinfected, draped, and given local anesthetized. The puncture site was chosen to be 2 cm below the inguinal ligament, and the catheter sheath was placed into the femoral artery using the Seldinger technique. First, the DSA technique helps with abdominal trunk and standard hepatic artery angiography to determine the tumor's location, size, and condition of the tumor. Once the tumor is understood, the catheter sheath is advanced deeper into the left or right hepatic artery or the vessel that feeds the tumor, 5‐fluorouracil (500 mg/m^2^) or oxaliplatin (100 mg/m^2^) was injected into the proper hepatic artery, and embolization was performed using different embolization materials. The embolization materials used were iodized oil and gelatin sponge cubes, or iodized oil only, which was entirely mixed with these chemotherapeutic drugs as an emulsion and injected. Because TACE was performed at different hospitals, embolization materials varied. Patients were asked to return to the hospital 4–8 weeks after embolization for follow‐up investigations, including routine blood tests, liver, and kidney function, coagulation function, AFP, and imaging. Imaging included abdominal enhanced CT, MRI, or chest X‐ray scans. The above procedures were performed by highly qualified attending physicians who received relevant interventional medicine training.

### 2.4 Stratification according to the initial maximum diameter of the tumor

The maximum tumor diameters were measured by enhanced CT or MRI before surgical resection or preoperative TACE in all patients. According to the maximum tumor diameter, all HCC patients were divided into the 5–9.9 cm group and the ≥10 cm group, which were then defined as Group A and Group B, respectively.^18^

### 2.5 Postoperative follow‐up and study endpoints

The reexamination frequency of all patients after the operation was once every 2–3 months in the first 6 months, once every 3–6 months in the following 18 months, and then once every 6–9 months if there was no recurrence. The postoperative follow‐up included liver biochemistry, routine blood tests, coagulation function, AFP, chest X‐ray or chest CT scans, abdominal B ultrasound, abdominal enhanced CT or MRI. Radiofrequency ablation, TACE, chemotherapy, molecular targeted therapy, surgical re‐resection, or liver transplantation were performed according to the recurrence and the patient's wishes when the patient was diagnosed with recurrence. Life‐supporting treatment was given to the end‐stage patient.

Study endpoints included complications within 30 days, recurrence‐free survival (RFS), and OS. Postoperative liver failure (PLF) was defined as serum TBIL >50 μoml/L and prothrombin activity (PTA) <50% on day 5 after hepatectomy,^45^ postoperative bile leakage was defined as ≥3 days after surgery with a bilirubin concentration in the drain exceeding three times the normal bilirubin concentration in plasma.^46^ OS was defined as the time from the date of surgery to the date of patient death or last follow‐up, and RFS was defined as the time from the date of surgery to the date of first postoperative tumor recurrence or last follow‐up. The cut‐off last date was July 1, 2021.

### 2.6 Statistical analysis

Continuous variables were expressed as median (range) or mean ± standard deviation (SD), categorical variables were reported as number (*n*) or percentages of patients (%). Continuous variables were compared by the Student's *t*‐test or Mann–Whitney *U*‐test. Categorical variables were compared by the χ^2^ test or Fisher's exact test. The survival curves of RFS and OS of patients who received or did not receive TACE before surgery were generated by the Kaplan–Meier method, and the log‐rank test was used to compare the differences. The Cox proportional hazard regression analyses were used to adjust for other prognostic factors associated with RFS and OS. All statistical analyses and visualizations of this study were obtained by R version 3.6.1 with the SVA. A *p* value <0.05 was considered statistically significant.

## 3 RESULTS

### 3.1 Baseline clinicopathological and postoperative complications

During the study period, 2560 HCC patients underwent radical HCC resection, of which 556 solitary HCC patients with diameter ≥5 cm were included in the study cohort. The baseline characteristics, clinicopathological features, and postoperative complications of the entire population are presented in Table 1.

**TABLE 1:** Comparison of clinicopathological characteristics and perioperative outcomes between patients with and without preoperative TACE in the total population

Variable		Overall (556)	Non‐TACE (*n* = 406)	TACE (*n* = 150)	*p*
Age (%)	<60 years	318 (57.2)	228 (56.2)	90 (60.0)	0.474
	≥60 years	238 (42.8)	178 (43.8)	60 (40.0)	
Gender (%)	Female	137 (24.6)	106 (26.1)	31 (20.7)	0.226
	Male	419 (75.4)	300 (73.9)	119 (79.3)	
HBV (%)	No	23 (4.1)	18 (4.4)	5 (3.3)	0.735
	Yes	533 (95.9)	388 (95.6)	145 (96.7)	
HCV (%)	No	548 (98.6)	400 (98.5)	148 (98.7)	1.000
	Yes	8 (1.4)	6 (1.5)	2 (1.3)	
Cirrhosis (%)	No	181 (32.6)	133 (32.8)	48 (32.0)	0.946
	Yes	375 (67.4)	273 (67.2)	102 (68.0)	
Child‐Pugh (%)	A	490 (88.1)	362 (89.2)	128 (85.3)	0.275
	B	66 (11.9)	44 (10.8)	22 (14.7)	
ALT (%)	<50 U/L	354 (63.7)	259 (63.8)	95 (63.3)	0.999
	≥50 U/L	202 (36.3)	147 (36.2)	55 (36.7)	
AST (%)	<40 U/L	198 (35.6)	145 (35.7)	53 (35.3)	1.000
	≥40 U/L	358 (64.4)	261 (64.3)	97 (64.7)	
GGT (%)	<45 U/L	116 (20.9)	85 (20.9)	31 (20.7)	1.000
	≥45 U/L	440 (79.1)	321 (79.1)	119 (79.3)	
ALP (%)	<125 U/L	420 (75.5)	306 (75.4)	114 (76.0)	0.966
	≥125 U/L	136 (24.5)	100 (24.6)	36 (24.0)	
Alb (%)	<35 g/L	94 (16.9)	65 (16.0)	29 (19.3)	0.423
	≥35 g/L	462 (83.1)	341 (84.0)	121 (80.7)	
TBIL (%)	<20.4 μmol/L	466 (83.8)	341 (84.0)	125 (83.3)	0.955
	≥20.4 μmol/L	90 (16.2)	65 (16.0)	25 (16.7)	
DBIL (%)	<6.8 μmol/L	446 (80.2)	327 (80.5)	119 (79.3)	0.843
	≥6.8 μmol/L	110 (19.8)	79 (19.5)	31 (20.7)	
CR (%)	<84 μmol/L	474 (85.3)	349 (86.0)	125 (83.3)	0.522
	≥84 μmol/L	82 (14.7)	57 (14.0)	25 (16.7)	
INR (%)	<1.15	379 (68.2)	280 (69.0)	99 (66.0)	0.573
	≥1.15	177 (31.8)	126 (31.0)	51 (34.0)	
PLT (%)	<100	160 (28.8)	115 (28.3)	45 (30.0)	0.778
	≥100	396 (71.2)	291 (71.7)	105 (70.0)	
AFP (%)	<400 μg/L	355 (63.8)	258 (63.5)	97 (64.7)	0.885
	≥400 μg/L	201 (36.2)	148 (36.5)	53 (35.3)	
Maximum tumor size	Mean ± SD	9.92 (2.48)	9.85 (2.52)	10.10 (2.39)	0.304
Size group (%)	Group A	241 (43.3)	169 (41.6)	72 (48.0)	0.211
	Group B	315 (56.7)	237 (58.4)	78 (52.0)	
Edmondson grade (%)	I + II	79 (14.2)	59 (14.5)	20 (13.3)	0.824
	III + IV	477 (85.8)	347 (85.5)	130 (86.7)	
MVI (%)	No	197 (35.4)	144 (35.5)	53 (35.3)	1.000
	Yes	359 (64.6)	262 (64.5)	97 (64.7)	
Satellite nodules	No	336 (60.4)	247 (60.8)	89 (59.3)	0.823
	Yes	220 (39.6)	159 (39.2)	61 (40.7)	
Tumor capsule (%)	Absent or partial	432 (77.7)	321 (79.1)	111 (74.0)	0.247
	Complete	124 (22.3)	85 (20.9)	39 (26.0)	
Type of liver resection	Non‐anatomical	339 (61.0)	246 (60.6)	93 (62.0)	0.838
	Anatomical	217 (39.0)	160 (39.4)	57 (38.0)	
Postoperative adjuvant TACE	No	280 (50.4)	205 (50.5)	75 (50.0)	0.994
	Yes	276 (49.6)	201 (49.5)	75 (50.0)	
Perioperative mortality	No	552 (99.3)	404 (99.5)	148 (98.7)	0.295
	Yes	4 (0.7)	2 (0.5)	2 (1.3)	
PLF (%)	No	534 (96.0)	388 (95.6)	146 (97.3)	0.482
	Yes	22 (4.0)	18 (4.4)	4 (2.7)	
Abdominal hemorrhage (%)	No	549 (98.7)	401 (98.8)	148 (98.7)	1.000
	Yes	7 (1.3)	5 (1.2)	2 (1.3)	
Bile leakage (%)	No	543 (97.7)	396 (97.5)	147 (98.0)	0.996
	Yes	13 (2.3)	10 (2.5)	3 (2.0)	
Incisional infection (%)	No	516 (92.8)	374 (92.1)	142 (94.7)	0.397
	Yes	40 (7.2)	32 (7.9)	8 (5.3)	
Organ/space infection (%)	No	524 (94.2)	381 (93.8)	143 (95.3)	0.642
	Yes	32 (5.8)	25 (6.2)	7 (4.7)	
Respiratory infection (%)	No	545 (98.0)	399 (98.3)	146 (97.3)	0.715
	Yes	11 (2.0)	7 (1.7)	4 (2.7)	
Pleural effusion (%)	No	495 (89.0)	365 (89.9)	130 (86.7)	0.352
	Yes	61 (11.0)	41 (10.1)9	20 (13.3)	
Ascites (%)	No	507 (91.2)	370 (91.1)	137 (91.3)	1.000
	Yes	49 (8.8)	36 (8.9)	13 (8.7)	
Other complications (%)	No	539 (96.9)	395 (97.3)	144 (96.0)	0.612
	Yes	17 (3.1)	11 (2.7)	6 (4.0)	

Abbreviations: AFP, alpha‐fetoprotein; Alb, albumin; ALP, alkaline phosphatase; ALT, alanine aminotransferase; AST, aspartate aminotransferase; CR, creatinine; DBIL, direct bilirubin; GGT, gamma‐glutamy transpeptidase; HBV, hepatitis B virus; HCV, hepatitis C virus; INR, international normalized ratio; PLF, postoperative liver failure; PLT, blood platelet; TACE, transcatheter arterial chemoembolization; TBIL, total bilirubin.

Out of the 556 HCC patients, 150 (27.0%) were treated with preoperative TACE and 406 (73.0%) were not treated with preoperative TACE. For patients who only had one TACE session, the median interval between the TACE and surgery was 5 weeks (range 4–8), and for patients who had multiple preoperative TACE sessions, the median interval between the last TACE and surgery was 4 weeks (range 3–6). There were no significant differences in age, sex, cirrhosis, tumor diameter, Child‐Pugh classification, MVI, pathological grade, postoperative complications, and other variables between the two groups (*p* > 0.05). The baseline characteristics, clinicopathological features, and postoperative complications of each subgroup are listed in Table 2.

**TABLE 2:** Comparison of clinicopathological characteristics and perioperative outcomes between patients with and without preoperative TACE in Group A and Group B

Variable		Group A (*n* = 315)			Group B (*n* = 241)		
		Non‐TACE (*n* = 237)	TACE (*n* = 78)	*p*	Non‐TACE (*n* = 169)	TACE (*n* = 72)	*p*
Age (%)	<65 years	132 (55.7)	50 (64.1)	0.241	96 (56.8)	40 (55.6)	0.970
	≥65 years	105 (44.3)	28 (35.9)		73 (43.2)	32 (44.4)	
Gender (%)	Female	59 (24.9)	17 (21.8)	0.687	47 (27.8)	14 (19.4)	0.228
	Male	178 (75.1)	61 (78.2)		122 (72.2)	58 (80.6)	
HBV (%)	No	9 (3.8)	0 (0.0)	0.176	9 (5.3)	5 (6.9)	0.849
	Yes	228 (96.2)	78 (100.0)		160 (94.7)	67 (93.1)	
HCV (%)	No	235 (99.2)	78 (100.0)	1.000	165 (97.6)	70 (97.2)	1.000
	Yes	2 (0.8)	0 (0.0)		4 (2.4)	2 (2.8)	
Cirrhosis (%)	No	75 (31.6)	32 (41.0)	0.168	58 (34.3)	16 (22.2)	0.087
	Yes	162 (68.4)	46 (59.0)		111 (65.7)	56 (77.8)	
Child‐Pugh (%)	A	214 (90.3)	66 (84.6)	0.239	148 (87.6)	62 (86.1)	0.920
	B	23 (9.7)	12 (15.4)		21 (12.4)	10 (13.9)	
ALT (%)	<50 U/L	157 (66.2)	49 (62.8)	0.679	102 (60.4)	46 (63.9)	0.710
	≥50 U/L	80 (33.8)	29 (37.2)		67 (39.6)	26 (36.1)	
AST (%)	<40 U/L	85 (35.9)	26 (33.3)	0.788	60 (35.5)	27 (37.5)	0.882
	≥40 U/L	152 (64.1)	52 (66.7)		109 (64.5)	45 (62.5)	
GGT (%)	<45 U/L	51 (21.5)	11 (14.1)	0.206	34 (20.1)	20 (27.8)	0.256
	≥45 U/L	186 (78.5)	67 (85.9)		135 (79.9)	52 (72.2)	
ALP (%)	<125 U/L	177 (74.7)	64 (82.1)	0.239	129 (76.3)	50 (69.4)	0.338
	≥125 U/L	60 (25.3)	14 (17.9)		40 (23.7)	22 (30.6)	
Alb (%)	<35 g/L	41 (17.3)	15 (19.2)	0.829	24 (14.2)	14 (19.4)	0.407
	≥35 g/L	196 (82.7)	63 (80.8)		145 (85.8)	58 (80.6)	
TBIL (%)	<20.4 μmol/L	201 (84.8)	65 (83.3)	0.895	140 (82.8)	60 (83.3)	1.000
	≥20.4 μmol/L	36 (15.2)	13 (16.7)		29 (17.2)	12 (16.7)	
DBIL (%)	<6.8 μmol/L	193 (81.4)	64 (82.1)	1.000	134 (79.3)	55 (76.4)	0.741
	≥6.8 μmol/L	44 (18.6)	14 (17.9)		35 (20.7)	17 (23.6)	
CR (%)	<84 μmol/L	203 (85.7)	66 (84.6)	0.968	146 (86.4)	59 (81.9)	0.491
	≥84 μmol/L	34 (14.3)	12 (15.4)		23 (13.6)	13 (18.1)	
INR (%)	<1.15	163 (68.8)	49 (62.8)	0.405	117 (69.2)	50 (69.4)	1.000
	≥1.15	74 (31.2)	29 (37.2)		52 (30.8)	22 (30.6)	
PLT (%)	<100	67 (28.3)	24 (30.8)	0.781	48 (28.4)	21 (29.2)	1.000
	≥100	170 (71.7)	54 (69.2)		121 (71.6)	51 (70.8)	
AFP (%)	<400 μg/L	161 (67.9)	52 (66.7)	0.946	97 (57.4)	45 (62.5)	0.552
	≥400 μg/L	76 (32.1)	26 (33.3)		72 (42.6)	27 (37.5)	
Maximum tumor size	Mean ± SD	8.06 (0.85)	8.21 (0.91)	0.183	12.37 (1.82)	12.41 (1.71)	0.366
Edmondson grade (%)	I + II	39 (16.5)	12 (15.4)	0.964	20 (11.8)	8 (11.1)	1.000
	III + IV	198 (83.5)	66 (84.6)		149 (88.2)	64 (88.9)	
MVI (%)	No	111 (46.8)	38 (48.7)	0.874	33 (19.5)	15 (20.8)	0.955
	Yes	126 (53.2)	40 (51.3)		136 (80.5)	57 (79.2)	
Satellite nodules	No	150 (63.3)	53 (67.9)	0.543	97 (57.4)	36 (50.0)	0.360
	Yes	87 (36.7)	25 (32.1)		72 (42.6)	36 (50.0)	
Tumor capsule (%)	Absent or partial	181 (76.4)	60 (76.9)	1.000	140 (82.8)	51 (70.8)	0.054
	Complete	56 (23.6)	18 (23.1)		29 (17.2)	21 (29.2)	
Type of liver resection	Non‐anatomical	147 (62.0)	47 (60.3)	0.885	99 (58.6)	46 (63.9)	0.531
	Anatomical	90 (38.0)	31 (39.7)		70 (41.4)	26 (36.1)	
Postoperative adjuvant TACE	No	120 (50.6)	45 (57.7)	0.341	85 (50.3)	30 (41.7)	0.277
	Yes	117 (49.4)	33 (42.3)		84 (49.7)	42 (58.3)	
Perioperative mortality	No	237 (100)	78 (100)		167 (98.8)	70 (97.2)	0.585
	Yes	0	0	1.000	2 (1.2)	2 (2.8)	
PLF, *n* (%)	No	228 (96.2)	76 (97.4)	0.874	160 (94.7)	70 (97.2)	0.596
	Yes	9 (3.8)	2 (2.6)		9 (5.3)	2 (2.8)	
Abdominal hemorrhage (%)	No	236 (99.6)	77 (98.7)	0.994	165 (97.6)	71 (98.6)	1.000
	Yes	1 (0.4)	1 (1.3)		4 (2.4)	1 (1.4)	
Bile leakage (%)	No	232 (97.9)	76 (97.4)	1.000	164 (97.0)	71 (98.6)	0.792
	Yes	5 (2.1)	2 (2.6)		5 (3.0)	1 (1.4)	
Incisional infection (%)	No	221 (93.2)	74 (94.9)	0.809	153 (90.5)	68 (94.4)	0.452
	Yes	16 (6.8)	4 (5.1)		16 (9.5)	4 (5.6)	
Organ/space infection (%)	No	224 (94.5)	76 (97.4)	0.457	157 (92.9)	67 (93.1)	1.000
	Yes	13 (5.5)	2 (2.6)		12 (7.1)	5 (6.9)	
Respiratory infection (%)	No	232 (97.9)	76 (97.4)	1.000	167 (98.8)	70 (97.2)	0.737
	Yes	5 (2.1)	2 (2.6)		2 (1.2)	2 (2.8)	
Pleural effusion (%)	No	216 (91.1)	67 (85.9)	0.266	149 (88.2)	63 (87.5)	1.000
	Yes	21 (8.9)	11 (14.1)		20 (11.8)	9 (12.5)	
Ascites (%)	No	210 (88.6)	73 (93.6)	0.295	160 (94.7)	64 (88.9)	0.183
	Yes	27 (11.4)	5 (6.4)		9 (5.3)	8 (11.1)	
Other complications (%)	No	233 (98.3)	75 (96.2)	0.497	162 (95.9)	69 (95.8)	1.000
	Yes	4 (1.7)	3 (3.8)		7 (4.1)	3 (4.2)	

Abbreviations: AFP, alpha‐fetoprotein; Alb, albumin; ALP, alkaline phosphatase; ALT, alanine aminotransferase; AST, aspartate aminotransferase; CR, creatinine; DBIL, direct bilirubin; GGT, gamma‐glutamy transpeptidase; HBV, hepatitis B virus; HCV, hepatitis C virus; INR, international normalized ratio; PLF, postoperative liver failure; PLT, blood platelet; TACE, transcatheter arterial chemoembolization; TBIL, total bilirubin.

### 3.2 The effects of preoperative TACE on the prognosis of HCC in two groups

**FIGURE 1:**
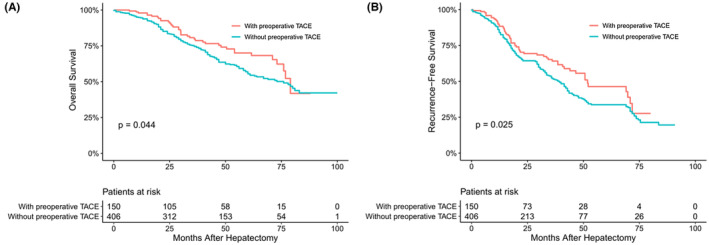
Overall (A) and recurrence‐free (B) survival curves of entire hepatocellular carcinoma patients with or without preoperative transcatheter arterial chemoembolization (TACE)

**FIGURE 2:**
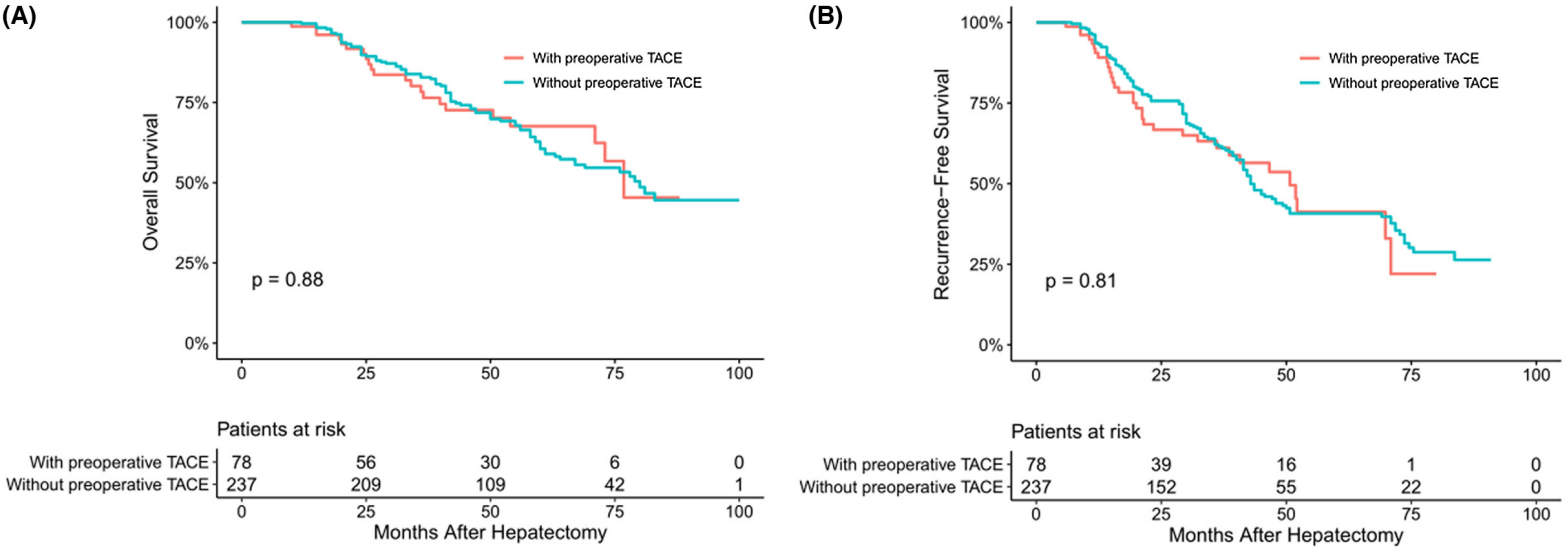
Overall (A) and recurrence‐free (B) survival curves of hepatocellular carcinoma patients in Group A with or without preoperative transcatheter arterial chemoembolization (TACE)

**FIGURE 3:**
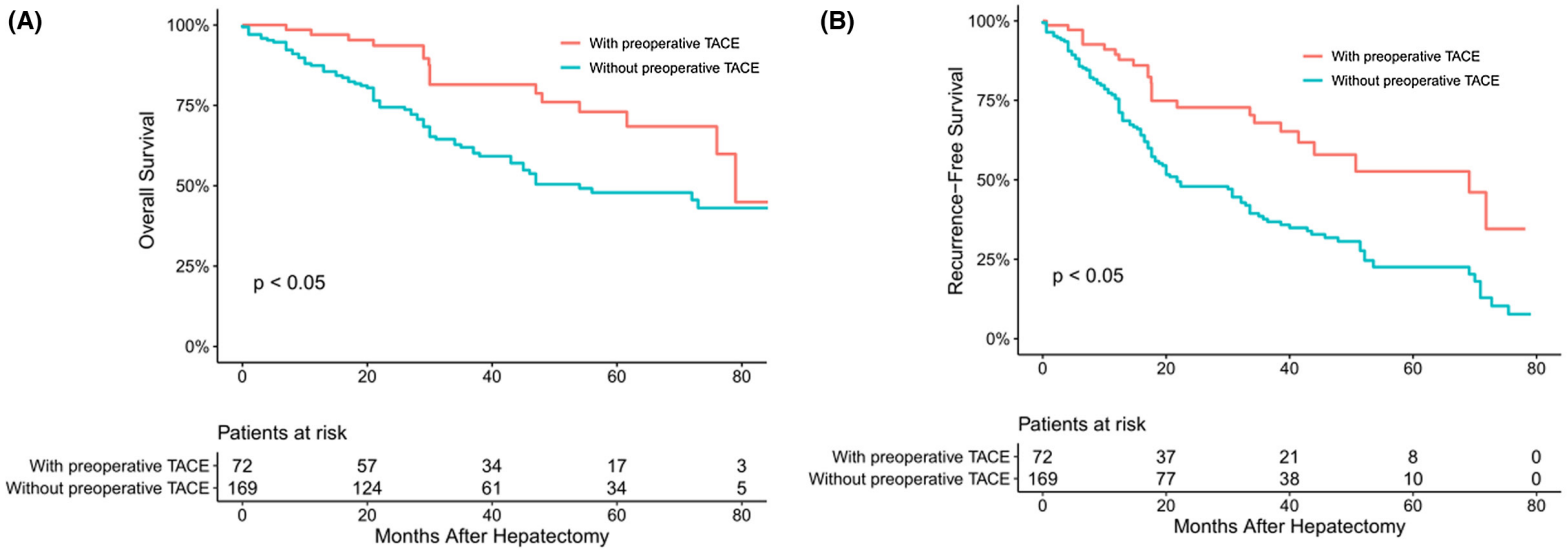
Overall (A) and recurrence‐free (B) survival curves of hepatocellular carcinoma patients in Group B with or without preoperative transcatheter arterial chemoembolization (TACE)

The median follow‐up time of the overall HCC population was 41 months. The mortality (25.3% vs. 39.4%, *p* < 0.05) and recurrence (38.0% vs. 58.4%, *p* < 0.05) rates of patients undergoing surgical resection with TACE were lower than those without TACE, showing statistical differences. The median OS and RFS of patients with TACE before surgery were 76 months and 37 months, respectively, longer than those without TACE (73 months and 32 months, respectively, *p* = 0.044 and 0.025) (Figure 1A,B). Then, we stratified according to the tumor diameter and found that there was no significant difference in OS and RFS between patients with TACE and those without TACE in Group A (*p* = 0.88 and *p* = 0.81, respectively) (Figure 2A,B). However, at Group B, the OS and RFS of patients with preoperative TACE were significantly better than those without preoperative TACE (*p* < 0.05 and *p* < 0.05, respectively) (Figure 3A,B).

### 3.3 Univariable and multivariable analyses of OS and RFS

Results of univariate and multivariate analyses for the entire study cohort's overall and recurrence‐free survivals are presented in Tables 3 and 4, respectively. Univariate and multivariate Cox‐regression analysis showed that preoperative TACE could reduce postoperative recurrence (HR = 0.658, 95% CI: 0.490–0.885; *p* = 0.006) and prolong the survival (HR = 0.673, 95% CI: 0.470–0.963; *p =* 0.030). In the study cohort of each subgroup, the univariate and multivariate analysis results were listed in Table 5; Tables S1–S3, respectively. At Group A, the result showed that preoperative TACE had no statistical significance with RFS (HR = 1.045, 95% CI: 0.71–1.538; *p* = 0.823) and OS (HR = 0.961, 95% CI: 0.601–1.537; *p* = 0.869). However, in the Group B, the result reported that preoperative TACE could improve OS (HR = 0.448, 95% CI: 0.260–0.773; *p* = 0.004).and RFS (HR = 0.419, 95% CI: 0.269–0.652; *p* < 0.005).

**TABLE 3:** Univariate and multivariate Cox‐regression analyses for overall survival in the total population

Variables	HR comparison	UV HR (95% CI)	UV *p*	MV HR (95% CI)	MV *p* *
Preoperative TACE	Yes versus no	0.696 (0.489–0.993)	0.045	0.673 (0.470–0.963)	0.030
Age	≥60 versus <60 years	1.339 (1.013–1.77)	0.040	1.434 (0.99–1.905)	0.065
Gender	Male versus female	1.321 (0.934–1.869)	0.116		
HBV	Yes versus no	1.588 (0.746–3.38)	0.230		
HCV	Yes versus no	0.567 (0.141–2.286)	0.425		
Cirrhosis	Yes versus no	0.95 (0.703–1.283)	0.737		
Child‐Pugh	B versus A	1.237 (0.793–1.929)	0.348		
ALT	≥50 versus <50 U/L	1.059 (0.796–1.409)	0.695		
AST	≥40 versus <40 U/L	1.096 (0.816–1.471)	0.543		
GGT	≥45 versus <45 U/L	1.222 (0.844–1.769)	0.288		
ALP	≥40 versus <40 U/L	0.871 (0.621–1.222)	0.424		
Alb	≥35 versus <35 g/L	0.82 (0.571–1.178)	0.284		
TBIL	≥20.4 versus <20.4 μmol/L	0.907 (0.615–1.338)	0.622		
DBIL	≥6.8 versus <6.8 μmol /L	0.982 (0.692–1.395)	0.919		
CR	≥80.4 versus <80.4 μmol /L	0.748 (0.488–1.146)	0.182		
INR	≥1.15 versus <1.15	0.899 (0.661–1.224)	0.500		
PLT	≥100 versus <100 × 10^9^/L	1.391 (0.992–1.95)	0.056		
AFP	≥400 versus <400 ng/L	2.026 (1.528–2.685)	0.000	1.878 (1.41–2.501)	0.000
Maximum tumor size	Group A versus Group B	0.685 (0.517–0.908)	0.008	0.766 (0.57–0.929)	0.045
Edmondson grade	III + IV versus. I + II	3.195 (1.955–5.224)	0.000	3.211 (1.959–5.261)	0.000
MVI	Yes versus no	1.979 (1.452–2.697)	0.000	1.824 (1.310–2.542)	0.000
Satellite nodules	Yes versus no	1.543 (1.322–2.996)	0.000	1.721 (1.112–2.673)	0.020
Tumor capsule	Complete versus incomplete	0.883 (0.639–1.221)	0.451		
Type of liver resection	Anatomical versus non‐anatomical	1.237 (0.424–2.342)	0.453		
Postoperative adjuvant TACE	Yes versus no	0.850 (0.632–1.768)	0.654		

Abbreviations: AFP, alpha‐fetoprotein; Alb, albumin; ALP, alkaline phosphatase; ALT, alanine aminotransferase; AST, aspartate aminotransferase; CI, confidence interval; CR, creatinine; DBIL, direct bilirubin; GGT, gamma‐glutamy transpeptidase; HBV, hepatitis B virus; HCV, hepatitis C virus; HR, hazard ratio; INR, international normalized ratio; MV, multivariable; PLT, blood platelet; TACE, transcatheter arterial chemoembolization; TBIL, total bilirubin; UV, univariable.

* Those variables found significant at *p* < 0.05 in univariable analyses were entered into multivariable Cox‐regression analyses.

**TABLE 4:** Univariate and multivariate Cox‐regression analyses for recurrence‐free survival in the total population

Variables	HR comparison	UV HR (95% CI)	UV *p*	MV HR (95% CI)	MV *p* *
Preoperative TACE	Yes versus no	0.719 (0.538–0.961)	0.026	0.658 (0.49–0.885)	0.006
Age	≥60 versus <60 years	1.092 (0.866–1.377)	0.456	1.434 (0.98–1.905)	0.073
Gender	Male versus female	1.115 (0.85–1.462)	0.432		
HBV	Yes versus no	1.108 (0.658–1.865)	0.699		
HCV	Yes versus no	0.936 (0.386–2.268)	0.884		
Cirrhosis	Yes versus no	1.008 (0.784–1.295)	0.951		
Child‐Pugh	B versus A	1.508 (1.071–2.121)	0.018	1.136 (0.8–1.612)	0.476
ALT	≥50 versus <50 U/L	1.086 (0.86–1.372)	0.486		
AST	≥40 versus <40 U/L	1.032 (0.812–1.312)	0.795		
GGT	≥45 versus <45 U/L	1.188 (0.88–1.604)	0.261		
ALP	≥40 versus <40 U/L	0.95 (0.725–1.247)	0.713		
Alb	≥35 versus <35 g/L	0.93 (0.68–1.271)	0.647		
TBIL	≥20.4 versus <20.4 μmol/L	0.869 (0.629–1.202)	0.397		
DBIL	≥6.8 versus <6.8 μmol /L	1.013 (0.761–1.349)	0.928		
CR	≥80.4 versus <80.4 μmol /L	0.877 (0.632–1.217)	0.434		
INR	≥1.15 versus <1.15	1.08 (0.845–1.379)	0.540		
PLT	≥ 100 versus <100 × 10^9^/L	1.059 (0.817–1.372)	0.663		
AFP	≥400 versus <400 ng/L	3.283 (2.595–4.153)	0.000	2.936 (2.303–3.743)	0.000
Maximum tumor size	Group A versus Group B	0.615 (0.488–0.775)	0.000	0.702 (0.551–0.895)	0.004
Edmondson grade	III + IV versus I + II	2.807 (1.94–4.062)	0.000	3.024 (2.075–4.407)	0.000
MVI	Yes versus no	2.522 (1.933–3.29)	0.000	1.982 (1.456–2.699)	0.000
Satellite nodules	Yes versus no	2.563 (1.322–3.235)	0.000	1.871 (1.345–2.677)	0.003
Tumor capsule	Complete versus incomplete	0.582 (0.433–0.783)	0.000	1.016 (0.726–1.423)	0.925
Type of liver resection	Anatomical versus non‐anatomical	1.048 (0.316–2.441)	0.753		
Postoperative adjuvant TACE	Yes versus no	0.651 (0.424–1.427)	0.435		

Abbreviations: AFP, alpha‐fetoprotein; Alb, albumin; ALP, alkaline phosphatase; ALT, alanine aminotransferase; AST, aspartate aminotransferase; CI, confidence interval; CR, creatinine; DBIL, direct bilirubin; GGT, gamma‐glutamy transpeptidase; HBV, hepatitis B virus; HCV, hepatitis C virus; HR, hazard ratio; INR, international normalized ratio; MV, multivariable; PLT, blood platelet; TACE, transcatheter arterial chemoembolization; TBIL, total bilirubin; UV, univariable.

* Those variables found significant at *p* < 0.05 in univariable analyses were entered into multivariable Cox‐regression analyses.

**TABLE 5:** Univariate and multivariate Cox‐regression analyses for overall survival in the Group A

Variables	HR comparison	UV HR (95% CI)	UV *p*	MV HR (95% CI)	MV *p* *
Preoperative TACE	Yes versus no	0.961 (0.601–1.537)	0.869		
Age	≥60 versus <60 years	1.182 (0.812–1.720)	0.384		
Gender	Male versus female	1.131 (0.719–1.778)	0.594		
HBV	Yes versus no	0.949 (0.349–2.579)	0.919		
HCV	Yes versus no	2.168 (0.301–15.623)	0.442		
Cirrhosis	Yes versus no	0.825 (0.558–1.220)	0.335		
Child‐Pugh	B versus A	2.197 (1.288–3.749)	0.004	1.786 (1.033–3.091)	0.038
ALT	≥50 versus <50 U/L	1.156 (0.789–1.694)	0.457		
AST	≥40 versus <40 U/L	1.152 (0.771–1.721)	0.489		
GGT	≥45 versus <45 U/L	1.149 (0.707–1.867)	0.574		
ALP	≥40 versus <40 U/L	1.036 (0.667–1.609)	0.876		
Alb	≥35 versus <35 g/L	0.786 (0.492–1.258)	0.316		
TBIL	≥20.4 versus <20.4 μmol/L	0.912 (0.537–1.551)	0.735		
DBIL	≥6.8 versus <6.8 μmol /L	1.109 (0.695–1.772)	0.663		
CR	≥80.4 versus <80.4 μmol /L	0.814 (0.465–1.427)	0.472		
INR	≥1.15 versus <1.15	1.124 (0.758–1.666)	0.561		
PLT	≥ 100 versus <100 × 10^9^/L	1.195 (0.778–1.835)	0.416		
AFP	≥400 versus <400 ng/L	1.771 (1.206–2.601)	0.004	1.517 (1.026–2.244)	0.037
Edmondson grade	III + IV versus I + II	2.615 (1.457–4.693)	0.001	2.524 (1.400–4.551)	0.002
MVI	Yes versus no	1.694 (1.158–2.477)	0.007	1.572 (1.065–2.316)	0.023
Satellite nodules	Yes versus no	1.563 (1.265–3.286)	0.000	1.844 (1.045–3.677)	0.003
Tumor capsule	Complete versus incomplete	0.844 (0.553–1.288)	0.433		
Type of liver resection	Anatomical versus non‐anatomical	1.246 (0.411–2.631)	0.651		
Postoperative adjuvant TACE	Yes versus no	0.543 (0.278–1.524)	0.543		

Abbreviations: AFP, alpha‐fetoprotein; Alb, albumin; ALP, alkaline phosphatase; ALT, alanine aminotransferase; AST, aspartate aminotransferase; CI, confidence interval; CR, creatinine; DBIL, direct bilirubin; GGT, gamma‐glutamy transpeptidase; HBV, hepatitis B virus; HCV, hepatitis C virus; HR, hazard ratio; INR, international normalized ratio; MV, multivariable; PLF, postoperative liver failure; PLT, blood platelet; TACE, transcatheter arterial chemoembolization; TBIL, total bilirubin; UV, univariable.

* Those variables found significant at *p* < 0.05 in univariable analyses were entered into multivariable Cox‐regression analyses.

### 3.4 Comparison of the clinicopathological features between Group A and Group B

The comparison of clinicopathological features between Group A and Group B is shown in Table S4.

Of the 315 patients in the Group A, 239 (75.9%) were male and 76 (24.1%) were female; 306 patients (97.1%) had chronic HBV infection and two patients (0.6%) were positive for hepatitis C virus RNA.

Among 241 patients with a maximum tumor diameter ≥10 cm (Group B), 180 (74.7%) patients were male, and 227 (94.2%) patients had chronic HBV infection. Patients who received preoperative TACE in the Group A less than those with preoperative TACE in the Group B (78/315 [24.8%] vs. 72/241 [29.9%]), but the difference was not statistically significant (*p* = 0.211). The proportion of patients with AFP ≥400 μg/L (41.1% vs. 32.4%, *p* = 0.043), MVI (80.1% vs. 52.7%, *p* < 0.001) and satellite nodules (44.8% vs. 35.6%, *p* = 0.034) in Group B was higher than that in Group A, while other clinicopathological indicators were not significantly different.

## 4 DISCUSSION

Transcatheter arterial chemoembolization is one of the most widely used non‐surgical therapeutic modalities for HCC. It mainly causes ischemic necrosis of the tumor by blocking the blood vessels feeding tumor, and at the same time delivers chemotherapy drugs through the artery to the target region to further promote tumor necrosis and tumor shrinkage. Recently, some researchers will consider it as a means of neoadjuvant therapy, the aim of which is mainly to improve the detection rate of latent intrahepatic metastasis or increase the resectability rate by decreasing the tumor diameter, and finally to improve the postoperative RFS and OS of HCC patients.^25^, ^35^, ^47^, ^48^, ^49^ However, it is controversial whether preoperative TACE is effective in reducing recurrence and prolonging survival.^17^, ^18^, ^19^, ^20^, ^21^, ^22^, ^23^, ^24^, ^25^, ^26^, ^27^, ^28^, ^29^, ^30^, ^31^, ^32^, ^33^, ^34^, ^35^, ^36^, ^37^, ^38^, ^39^, ^41^, ^50^

Interestingly, we found that the HCC population included in studies against preoperative adjuvant TACE generally had smaller tumor diameters (<5 cm), and that patients with preoperative TACE had much larger tumor diameters than those without preoperative TACE.^28^, ^29^, ^30^, ^31^, ^32^, ^33^, ^34^, ^35^, ^36^, ^37^, ^38^, ^39^, ^40^, ^41^ Therefore, the validity of their conclusions remained questionable, although they performed a propensity matched score (PSM) analysis.^31^, ^33^, ^35^, ^36^ In contrast, studies asserting that preoperative TACE was valuable usually included patients with LHCC (>5 cm) with more balanced clinicopathological data.^17^, ^18^, ^20^, ^21^, ^25^ So, HCC patients with a maximum tumor diameter of 5 cm or more were selected as our study population.

Some reports suggest that preoperative TACE may only be significant in patients with an excessively large tumor diameter,^17^, ^18^, ^19^, ^20^, ^21^, ^25^, ^26^ especially for patients with tumor diameter ≥10 cm.^18^ We then divided the population into Groups A (5–9.9 cm) and B (≥10 cm) and explored the efficacy of preoperative TACE in each subgroup of the HCC population separately.

The results of this study showed that preoperative TACE benefited HCC patients and improved their RFS and OS in the overall population. However, after stratification, it was clear that the benefit was only significant for patients with tumor diameters ≥10 cm. In recent years, two studies on preoperative TACE from the same medical center, they just will be different in the setting of the inclusion criteria, and then the conclusion is different, and Zhou et al. included the inclusion criterion of tumor diameter ≥5 cm, which concluded that preoperative TACE had no effect on RFS and OS of patient.^39^ Whereas, Li et al. set the cut‐off value of tumor diameter at 10 cm, their conclusion was quite different.^18^ This latter study illustrated that the benefit population of preoperative TACE might be patients with huge HCC (≥10 cm), which is consistent with our current results.

We speculate that the possible reason for this stratification effect is that the corresponding tumor vascularization of Group B is more severe. Since the degree of tumor vascularization is positively correlated with the effectiveness of TACE, preoperative TACE could play a more critical role in Group B and ultimately benefit the survival of patients.^19^, ^51^, ^52^ Second, as far as the tumor's biological characteristics are concerned, AFP level, MVI, and satellite nodules were higher in the Group B than in the Group A (*p* < 0.05), reflecting that the tumor biological characteristics of Group B were more aggressive. In other words, preoperative TACE could effectively inhibit the growth and proliferation of highly invasive HCC. Toshiya et al. suggested that preoperative TACE may be more suitable for more aggressive tumor populations.^22^, ^53^ Based on the above lines, we have reason to believe that for patients with solitary HCC with a diameter of ≥10 cm, preoperative TACE can improve the survival prognosis of HCC patients.

In terms of perioperative complications and 30‐day mortality, previous studies have shown that preoperative TACE could increase the intraoperative difficulty and perioperative complications.^26^, ^36^, ^38^ In our study, this view is not tenable, which is consistent with the result of Li et al.^18^, ^54^ The surgical procedure does have a small portion of patients with necrosis tumors adherent to the surrounding tissues. But our chief surgeons are experienced and can completely overcome the adhesions caused by TACE. Again, it has been reported that as long as the interval between preoperative TACE and the operation is long enough, the negative impact of preoperative TACE on operation can be controllable.^18^, ^54^ In our study, the interval is at least 4 weeks. Therefore, as long as the patients are appropriately managed during perioperative period, the obstruction of TACE to surgery can be eliminated.

The results of this study revealed that tumor diameter ≥10 cm, AFP ≥ 400 μg/L, MVI, Edmondson grade, PLT level, and satellite nodules were independent risk factors for postoperative OS and RFS, which were also confirmed by previous studies.^18^, ^55^, ^56^, ^57^, ^58^, ^59^, ^60^, ^61^, ^62^

Our research has limitations. First, our study was a multicenter retrospective study that did not have a uniform standard for preoperative TACE. Second, considering that this study is a multi‐center study, the embolic materials and chemotherapeutic drugs used in each center are different. Third, most of the people we include are infected with HBV, while the majority of HCC patients in western countries are caused by factors such as HCV or alcohol. This study may not be suitable for western populations.

In conclusion, our study demonstrated that preoperative TACE is a safe neoadjuvant that does not increase perioperative complications and mortality. There was a stratification effect on the efficacy of preoperative TACE, and the beneficiary population is HCC patients with tumor diameter ≥10 cm. This study provides further guidance for the treatment of patients with large and huge solitary HCC to avoid unnecessary preoperative TACE.

## AUTHOR CONTRIBUTIONS

**Ali Mo:** Conceptualization (equal). **Qiao Zhang:** Data curation (equal). **Feng Xia:** Conceptualization (equal); data curation (equal). **Zhiyuan Huang:** Resources (equal); software (equal). **Shasha Peng:** Supervision (equal); validation (equal). **Wenjing Cao:** Supervision (equal); validation (equal). **Hongliang Mei:** Data curation (equal); software (equal). **Li Ren:** Conceptualization (equal); resources (equal). **Yang Su:** Investigation (equal); methodology (equal). **Hengyi Gao:** Writing – original draft (equal); writing – review and editing (equal). **Weiqiang Chen:** Resources (equal); software (equal).

## ETHICS STATEMENT

The ethics committees of the six medical centers approved the study, and the study complied with the Helsinki Declaration and local laws.

## INFORMED CONSENT

The patient provided informed consent, which was registered in the medical record.

## Supporting information


Appendix S1.

## Data Availability

The datasets used and analyzed during the current study are available from the corresponding author on reasonable request.
