# Supplementary material for: Preoperative transcatheter arterial chemoembolization and prognosis of patients with solitary large hepatocellular carcinomas (≥5 cm): Multicenter retrospective study
Source: Cancer Med. 2022 Dec 20;12(7):7734–47. doi: 10.1002/cam4.5529 (PMC10134378; doi:10.1002/cam4.5529)
Supplement: Supplementary file 1 — Appendix S1. [file CAM4-12-7734-s001.docx]

**eTable 1** Univariate and multivariate Cox-regression analyses for recurrence-free survival in the Group A

| Variables | HR comparison | UV HR (95% *CI*) | | UV p | MV HR (95% *CI*) | MV p* |
| --- | --- | --- | --- | --- | --- | --- |
| Preoperative TACE | Yes vs. no | | 1.045(0.71-1.538) | 0.823 |  |  |
| Age | ≥60 vs＜60 years | | 0.994(0.724-1.365) | 0.970 |  |  |
| Gender | Male vs. female | | 0.928(0.648-1.329) | 0.684 |  |  |
| HBV | Yes vs. no | | 1.407(0.521-3.801) | 0.500 |  |  |
| HCV | Yes vs. no | | 1.621(0.226-11.636) | 0.631 |  |  |
| Cirrhosis | Yes vs. no | | 0.935(0.669-1.306) | 0.692 |  |  |
| Child Pugh | B vs A | | 1.841(1.136-2.983) | 0.013 | 1.363(0.828-2.243) | 0.223 |
| ALT | ≥50 vs＜50 U/L | | 1.131(0.822-1.556) | 0.450 |  |  |
| AST | ≥40 vs＜40 U/L | | 1.033(0.743-1.436) | 0.846 |  |  |
| GGT | ≥45 vs＜45 U/L | | 1.056(0.713-1.566) | 0.785 |  |  |
| ALP | ≥40 vs＜40 U/L | | 0.987(0.68-1.432) | 0.946 |  |  |
| Alb | ≥35 vs＜35 g/L | | 0.892(0.591-1.344) | 0.584 |  |  |
| TBIL | ≥20.4 vs＜20.4umol/L | | 0.772(0.483-1.234) | 0.280 |  |  |
| DBIL | ≥6.8 vs＜6.8umol /L | | 0.915(0.604-1.386) | 0.676 |  |  |
| CR | ≥80.4 vs＜80.4umol /L | | 0.945(0.607-1.471) | 0.801 |  |  |
| INR | ≥1.15 vs＜1.15 | | 1.24(0.897-1.714) | 0.194 |  |  |
| PLT | ≥ 100 vs＜100 × 10^9^/L | | 1.097(0.772-1.557) | 0.606 |  |  |
| AFP | ≥400 vs＜400ng/L | | 2.715(1.976-3.729) | 0.000 | 2.283(1.65-3.158) | 0.000 |
| Edmondson Grade | III+IV vs I+II | | 3.129(1.881-5.204) | 0.000 | 2.944(1.761-4.921) | 0.000 |
| MVI | Yes vs. no | | 2.154(1.558-2.977) | 0.000 | 1.862(1.296-2.677) | 0.001 |
| Satellite nodules | Yes vs. no | | 1.563(1.265-3.286) | 0.000 | 1.844(1.045-3.677) | 0.003 |
| Tumor capsule | Complete vs. incomplete | | 0.571(0.388-0.84) | 0.004 | 0.837(0.542-1.291) | 0.421 |
| Type of liver resection | Anatomical vs. non-anatomical | | 1.032(0.349-3.678) | 0.452 |  |  |
| Postoperative adjuvant TACE | Yes vs. no | | 0.543(0.138-1.214) | 0.958 |  |  |

*TACE* transcatheter arterial chemoembolization*，AST* aspartate aminotransferase, *ALT* alanine aminotransferase, *GGT* gamma-glutamy transpeptidase, *ALP* alkaline phosphatase, *Alb* albumin, *TBIL* total bilirubin, *DBIL* direct bilirubin, *CR* creatinine, INR international normalized ratio, *PLT* blood platelet, *AFP* alpha-fetoprotein, *PLF* postoperative liver failure, *HBV* hepatitis B virus, *HCV* hepatitis C virus, *CI* confidence interval, *HR* hazard ratio, *UV* univariable, *MV* multivariable

*Those variables found significant at *p* < 0.05 in univariable analyses were entered into multivariable Cox-regression analyses

**eTable 2** Univariate and multivariate Cox-regression analyses for overall survival in the Group B

| Variables | HR comparison | UV HR (95% *CI*) | UV p | MV HR (95% *CI*) | MV p* |
| --- | --- | --- | --- | --- | --- |
| Preoperative TACE | Yes vs. no | 0.453(0.263-0.779) | 0.004 | 0.448(0.26-0.773) | 0.004 |
| Age | ≥60 vs＜60 years | 1.553(1.021-2.363) | 0.040 | 1.46(0.952-2.24) | 0.083 |
| Gender | Male vs. female | 1.63(0.948-2.802) | 0.077 |  |  |
| HBV | Yes vs. no | 2.737(0.863-8.682) | 0.087 |  |  |
| HCV | Yes vs. no | 0.292(0.041-2.099) | 0.221 |  |  |
| Cirrhosis | Yes vs. no | 1.12(0.695-1.804) | 0.643 |  |  |
| Child Pugh | B vs A | 0.564(0.246-1.293) | 0.176 |  |  |
| ALT | ≥50 vs＜50 U/L | 0.913(0.594-1.404) | 0.680 |  |  |
| AST | ≥40 vs＜40 U/L | 1.052(0.681-1.625) | 0.819 |  |  |
| GGT | ≥45 vs＜45 U/L | 1.372(0.774-2.431) | 0.279 |  |  |
| ALP | ≥40 vs＜40 U/L | 0.676(0.398-1.147) | 0.147 |  |  |
| Alb | ≥35 vs＜35 g/L | 0.831(0.469-1.474) | 0.527 |  |  |
| TBIL | ≥20.4 vs＜20.4umol/L | 0.912(0.514-1.618) | 0.753 |  |  |
| DBIL | ≥6.8 vs＜6.8umol /L | 0.822(0.484-1.398) | 0.469 |  |  |
| CR | ≥80.4 vs＜80.4umol /L | 0.684(0.353-1.328) | 0.263 |  |  |
| INR | ≥1.15 vs＜1.15 | 0.647(0.389-1.076) | 0.093 |  |  |
| PLT | ≥ 100 vs＜100 × 10^9^/L | 1.77(1.015-3.089) | 0.044 | 2.051(1.172-3.591) | 0.012 |
| AFP | ≥400 vs＜400ng/L | 2.249(1.474-3.431) | 0.000 | 2.091(1.36-3.214) | 0.001 |
| Edmondson Grade | III+IV vs I+II | 4.556(1.798-11.544) | 0.001 | 4.555(1.796-11.557) | 0.001 |
| MVI | Yes vs. no | 2.512(1.298-4.860) | 0.006 | 2.557(1.302-5.021) | 0.006 |
| Satellite nodules | Yes vs. no | 1.453(1.274-3.883) | 0.000 | 1.741(1.451-2.757) | 0.001 |
| Tumor capsule | Complete vs. incomplete | 0.956(0.575-1.591) | 0.863 |  |  |
| Type of liver resection | Anatomical vs. non-anatomical | 1.152(0.491-2.768) | 0.531 |  |  |
| Postoperative adjuvant TACE | Yes vs. no | 0.443(0.356-1.247) | 0.676 |  |  |

*TACE* transcatheter arterial chemoembolization*，AST* aspartate aminotransferase, *ALT* alanine aminotransferase, *GGT* gamma-glutamy transpeptidase, *ALP* alkaline phosphatase, *Alb* albumin, *TBIL* total bilirubin, *DBIL* direct bilirubin, *CR* creatinine, INR international normalized ratio, *PLT* blood platelet, *AFP* alpha-fetoprotein, *PLF* postoperative liver failure, *HBV* hepatitis B virus, *HCV* hepatitis C virus, *CI* confidence interval, *HR* hazard ratio, *UV* univariable, *MV* multivariable

*Those variables found significant at *p* < 0.05 in univariable analyses were entered into multivariable Cox-regression analyses

**eTable 3** Univariate and multivariate Cox-regression analyses for recurrence-free survival in the Group B

| Variables | HR comparison | UV HR (95% *CI*) | UV p | MV HR (95% *CI*) | MV p* |
| --- | --- | --- | --- | --- | --- |
| Preoperative TACE | Yes vs. no | 0.425(0.273-0.662) | 0.000 | 0.419(0.269-0.652) | 0.000 |
| Age | ≥60 vs＜60 years | 1.242(0.883-1.747) | 0.213 |  |  |
| Gender | Male vs. female | 1.436(0.947-2.178) | 0.088 |  |  |
| HBV | Yes vs. no | 1.165(0.626-2.169) | 0.629 |  |  |
| HCV | Yes vs. no | 0.677(0.249-1.842) | 0.445 |  |  |
| Cirrhosis | Yes vs. no | 1.054(0.718-1.546) | 0.789 |  |  |
| Child Pugh | B vs A | 1.2(0.738-1.951) | 0.463 |  |  |
| ALT | ≥50 vs＜50 U/L | 0.969(0.688-1.366) | 0.857 |  |  |
| AST | ≥40 vs＜40 U/L | 1.052(0.741-1.493) | 0.777 |  |  |
| GGT | ≥45 vs＜45 U/L | 1.418(0.889-2.26) | 0.142 |  |  |
| ALP | ≥40 vs＜40 U/L | 0.889(0.597-1.323) | 0.561 |  |  |
| Alb | ≥35 vs＜35 g/L | 0.915(0.562-1.490) | 0.722 |  |  |
| TBIL | ≥20.4 vs＜20.4umol/L | 0.998(0.636-1.566) | 0.994 |  |  |
| DBIL | ≥6.8 vs＜6.8umol /L | 1.089(0.731-1.622) | 0.676 |  |  |
| CR | ≥80.4 vs＜80.4umol /L | 0.771(0.471-1.262) | 0.301 |  |  |
| INR | ≥1.15 vs＜1.15 | 0.926(0.634-1.354) | 0.692 |  |  |
| PLT | ≥ 100 vs＜100 × 10^9^/L | 0.982(0.667-1.447) | 0.928 |  |  |
| AFP | ≥400 vs＜400ng/L | 3.815(2.665-5.462) | 0.000 | 3.547(2.458-5.116) | 0.000 |
| Edmondson Grade | III+IV vs I+II | 2.653(1.513-4.652) | 0.001 | 3.069(1.772-5.317) | 0.000 |
| MVI | Yes vs. no | 3.012(1.728-5.248) | 0.000 | 2.202(1.250-3.877) | 0.006 |
| Satellite nodules | Yes vs. no | 1.526(1.148-3.723) | 0.000 | 1.942(1.571-2.556) | 0.007 |
| Tumor capsule | Complete vs. incomplete | 0.646(0.405-1.030) | 0.067 |  |  |
| Type of liver resection | Anatomical vs. non-anatomical | 1.521(0.491-5.768) | 0.711 |  |  |
| Postoperative adjuvant TACE | Yes vs. no | 1.443(0.353-1.997) | 0.469 |  |  |

*TACE* transcatheter arterial chemoembolization*，AST* aspartate aminotransferase, *ALT* alanine aminotransferase, *GGT* gamma-glutamy transpeptidase, *ALP* alkaline phosphatase, *Alb* albumin, *TBIL* total bilirubin, *DBIL* direct bilirubin, *CR* creatinine, INR international normalized ratio, *PLT* blood platelet, *AFP* alpha-fetoprotein, *PLF* postoperative liver failure, *HBV* hepatitis B virus, *HCV* hepatitis C virus, *CI* confidence interval, *HR* hazard ratio, *UV* univariable, *MV* multivariable

*Those variables found significant at *p* < 0.05 in univariable analyses were entered into multivariable Cox-regression analyses

**eTable 4 Comparison of clinicopathological characteristics and perioperative outcomes in Groups A and Groups B**

| **Variable** |  | **Overall (556)** | **Group A (n=315)** | **Group B (n=241)** | ***p*** |
| --- | --- | --- | --- | --- | --- |
| Age (%) | <60years | 318 (57.2) | 182 (57.8) | 136 (56.4) | 0.817 |
|  | ≥60years | 238 (42.8) | 133 (42.2) | 105 (43.6) |  |
| Gender (%) | Female | 137 (24.6) | 76 (24.1) | 61 (25.3) | 0.824 |
|  | Male | 419 (75.4) | 239 (75.9) | 180 (74.7) |  |
| HBV (%) | No | 23 (4.1) | 9 (2.9) | 14 (5.8) | 0.129 |
|  | Yes | 533 (95.9) | 306 (97.1) | 227 (94.2) |  |
| HCV (%) | No | 548 (98.6) | 313 (99.4) | 235 (97.5) | 0.144 |
|  | Yes | 8 (1.4) | 2 (0.6) | 6 (2.5) |  |
| Cirrhosis (%) | No | 181 (32.6) | 107 (34.0) | 74 (30.7) | 0.47 |
|  | Yes | 375 (67.4) | 208 (66.0) | 167 (69.3) |  |
| Child Pugh (%) | A | 490 (88.1) | 280 (88.9) | 210 (87.1) | 0.617 |
|  | B | 66 (11.9) | 35 (11.1) | 31 (12.9) |  |
| ALT (%) | <50U/L | 354 (63.7) | 206 (65.4) | 148 (61.4) | 0.379 |
|  | ≥50U/L | 202 (36.3) | 109 (34.6) | 93 (38.6) |  |
| AST (%) | <40U/L | 198 (35.6) | 111 (35.2) | 87 (36.1) | 0.904 |
|  | ≥40U/L | 358 (64.4) | 204 (64.8) | 154 (63.9) |  |
| GGT (%) | <45U/L | 116 (20.9) | 62 (19.7) | 54 (22.4) | 0.498 |
|  | ≥45U/L | 440 (79.1) | 253 (80.3) | 187 (77.6) |  |
| ALP (%) | <125U/L | 420 (75.5) | 241 (76.5) | 179 (74.3) | 0.612 |
|  | ≥125U/L | 136 (24.5) | 74 (23.5) | 62 (25.7) |  |
| Alb (%) | <35g/l | 94 (16.9) | 56 (17.8) | 38 (15.8) | 0.608 |
|  | ≥35g/l | 462 (83.1) | 259 (82.2) | 203 (84.2) |  |
| TBIL (%) | <20.4umol/L | 466 (83.8) | 266 (84.4) | 200 (83.0) | 0.729 |
|  | ≥20.4umol/L | 90 (16.2) | 49 (15.6) | 41 (17.0) |  |
| DBIL (%) | <6.8umol/L | 446 (80.2) | 257 (81.6) | 189 (78.4) | 0.412 |
|  | ≥6.8umol/L | 110 (19.8) | 58 (18.4) | 52 (21.6) |  |
| CR (%) | <84umol/L | 474 (85.3) | 269 (85.4) | 205 (85.1) | 1 |
|  | ≥84umol/L | 82 (14.7) | 46 (14.6) | 36 (14.9) |  |
| INR (%) | <1.15 | 379 (68.2) | 212 (67.3) | 167 (69.3) | 0.683 |
|  | ≥1.15 | 177 (31.8) | 103 (32.7) | 74 (30.7) |  |
| PLT (%) | <100 | 160 (28.8) | 91 (28.9) | 69 (28.6) | 1 |
|  | ≥100 | 396 (71.2) | 224 (71.1) | 172 (71.4) |  |
| AFP (%) | <400ug/L | 355 (63.8) | 213 (67.6) | 142 (58.9) | 0.043 |
|  | ≥400ug/L | 201 (36.2) | 102 (32.4) | 99 (41.1) |  |
| Maximum tumor size | Mean ± SD | 9.92 (2.48) | 8.09 (0.87) | 12.30 (1.79) | <0.001 |
| Preoperative TACE | Non-TACE | 406 (73.0) | 237 (75.2) | 169 (70.1) | 0.211 |
|  | TACE | 150 (27.0) | 78 (24.8) | 72 (29.9) |  |
| Edmondson Grade (%) | I+II | 79 (14.2) | 51 (16.2) | 28 (11.6) | 0.159 |
|  | III+IV | 477 (85.8) | 264 (83.8) | 213 (88.4) |  |
| MVI (%) | No | 197 (35.4) | 149 (47.3) | 48 (19.9) | <0.001 |
|  | Yes | 359 (64.6) | 166 (52.7) | 193 (80.1) |  |
| Satellite nodules | No | 336 (60.4) | 203 (64.4) | 133 (55.2) | 0.034 |
|  | Yes | 220 (39.6) | 112 (35.6) | 108 (44.8) |  |
| Tumor capsule (%) | Absent or Partial | 432 (77.7) | 241 (76.5) | 191 (79.3) | 0.504 |
|  | Complete | 124 (22.3) | 74 (23.5) | 50 (20.7) |  |
| Type of liver resection | Non-anatomical | 339 (61.0) | 194 (61.6) | 145 (60.2) | 0.800 |
|  | Anatomical | 217 (39.0) | 121 (38.4) | 96 (39.8) |  |
| Postoperative adjuvant TACE | No | 280 (50.4) | 165 (52.4) | 115 (47.7) | 0.315 |
|  | Yes | 276 (49.6) | 150 (47.6) | 126 (52.3) |  |
| PLF (%) | No | 534 (96.0) | 304 (96.5) | 230 (95.4) | 0.672 |
|  | Yes | 22 (4.0) | 11 (3.5) | 11 (4.6) |  |
| Abdominal hemorrhage (%) | No | 549 (98.7) | 313 (99.4) | 236 (97.9) | 0.261 |
|  | Yes | 7 (1.3) | 2 (0.6) | 5 (2.1) |  |
| Bile leakage (%) | No | 543 (97.7) | 308 (97.8) | 235 (97.5) | 1 |
|  | Yes | 13 (2.3) | 7 (2.2) | 6 (2.5) |  |
| Incisional infection (%) | No | 516 (92.8) | 295 (93.7) | 221 (91.7) | 0.474 |
|  | Yes | 40 (7.2) | 20 (6.3) | 20 (8.3) |  |
| Organ/space infection (%) | No | 524 (94.2) | 300 (95.2) | 224 (92.9) | 0.334 |
|  | Yes | 32 (5.8) | 15 (4.8) | 17 (7.1) |  |
| Respiratory infection (%) | No | 545 (98.0) | 308 (97.8) | 237 (98.3) | 0.869 |
|  | Yes | 11 (2.0) | 7 (2.2) | 4 (1.7) |  |
| Pleural effusion (%) | No | 495 (89.0) | 283 (89.8) | 212 (88.0) | 0.573 |
|  | Yes | 61 (11.0) | 32 (10.2) | 29 (12.0) |  |
| Ascites (%) | No | 507 (91.2) | 283 (89.8) | 224 (92.9) | 0.259 |
|  | Yes | 49 (8.8) | 32 (10.2) | 17 (7.1) |  |
| Other complications (%) | No | 539 (96.9) | 308 (97.8) | 231 (95.9) | 0.289 |
|  | Yes | 17 (3.1) | 7 (2.2) | 10 (4.1) |  |

*TACE* transcatheter arterial chemoembolization*, AST* aspartate aminotransferase, *ALT* alanine aminotransferase, *GGT* gamma-glutamy transpeptidase, *ALP* alkaline phosphatase, *Alb* albumin, *TBIL* total bilirubin, *DBIL* direct bilirubin, *CR* creatinine, INR international normalized ratio, *PLT* blood platelet, *AFP* alpha-fetoprotein, *PLF* postoperative liver failure, *HBV* hepatitis B virus, *HCV* hepatitis C virus
